# Retraction Note: Pre-activation of mesenchymal stem cells with TNF-α, IL-1β and nitric oxide enhances its paracrine effects on radiation-induced intestinal injury

**DOI:** 10.1038/s41598-025-91160-3

**Published:** 2025-02-25

**Authors:** Hao Chen, Xiao-Hui Min, Qi-Yi Wang, Felix W. Leung, Liu Shi, Yu Zhou, Tao Yu, Chuan-Ming Wang, Geng An, Wei-Hong Sha, Qi-Kui Chen

**Affiliations:** 1 Department of Gastroenterology, Sun Yat-SenMemorial Hospital, Sun Yat-Sen University, Guangzhou, People’s Republic of China; 2 Department of Gastroenterology, Guangdong General Hospital and Guangdong Academy of Medical Sciences, Guangzhou, People’s Republic of China; 3 Division of Gastroenterology, David Geffen School of Medicine at University of California, Los Angeles, USA; 4 Department of Gastroenterology, Ganzhou People’s Hospital, Ganzhou, People’s Republic of China; 5 Department of Gastroenterology, Affiliated Hospital of Guangdong Medical College, Zhanjian, People’s Republic of China; 6 Department of Neurology, Shenzhen Sixth People’s Hospital, Shenzhen, People’s Republic of China; 7 Department of Reproductive Medicine Center, The Third Affiliated Hospital of Guangzhou Medical University, Guangzhou, People’s Republic of China

Retraction of: *Scientific Reports*, 10.1038/srep08718, published on 3 March 2015

The Editors have retracted this Article.

A similarity was detected between the fourth image in Fig. 4A of this Article, and a fourth image in the second row of Fig. 1F of another paper^1^, where this image is described differently. Authors were not able to satisfactorily explain this. Furthermore, there appears to be a duplication and rotation between β-actin in Supplementary Fig. 5C and β-tubulin in Fig. 8D. The Editors were not able to verify that the data provided by Authors on request was original and, additionally, for Supplementary Fig. 5C and Fig. 8D, the data did not match the published figures. The Editors therefore have lost confidence in the data and conclusions of this Article.

The Authors have not responded to correspondence from the Publisher regarding this retraction.
